# Supplementary material for: The effects of genital myiasis on the diversity of the vaginal microbiota in female Bactrian camels
Source: BMC Vet Res. 2022 Mar 5;18:87. doi: 10.1186/s12917-022-03189-5 (PMC8897907; doi:10.1186/s12917-022-03189-5)
Supplement: Supplementary file 5 — Additional file 5. [file 12917_2022_3189_MOESM5_ESM.zip › MPL201709200_16s_yy/Treat1/css/js_css/jsPDF-master.zip!/jsPDF-master/examples/html2pdf/lists.html]

Html2Pdf


# Ordered And Unordered Lists

## Implemented

1. OL Item (default)
2. OL Item (decimal)
3. OL Item (upper-alpha)
4. OL Item (lower-alpha)
5. OL Item (upper-roman)
6. OL Item (lower-roman)
7. OL Item (none)

- UL Item (default)
- UL Item (circle)
- UL Item (square)
- UL Item (disc)
- OL Item (none)

## Not Implemented

```
    upper-latin
    lower-latin
    armenian
    cjk-ideographic
    decimal-leading-zero
    georgian
    hebrew
    hiragana
    hiragana-iroha
    katakana
    katakana-iroha
```
